# Retraction: Stereochemical Insignificance Discovered in *Acinetobacter baumannii* Quorum Sensing

**DOI:** 10.1371/annotation/1345f9b0-016e-4123-9e72-6ccc4fa17ba2

**Published:** 2013-05-10

**Authors:** Amanda L. Garner, Sook Kyung Kim, Jie Zhu, Anjali Kumari Struss, Richard Watkins, Brent D. Feske, Gunnar F. Kaufmann, Kim D. Janda

The authors request the retraction of the manuscript, “Stereochemical Insignificance Discovered in Acinetobacter baumannii Quorum Sensing,” as the main conclusion reporting a case of stereochemical insignificance in A. baumannii is not supported by more recent data generated by our and another group.

Subsequent to our publication, another laboratory reported the synthesis and biological evaluation of the acylhomoserine lactones (AHLs), N-((R)-3-hydroxydodecanoyl)-L-homoserine lactone (3a) and N-((S)-3-hydroxydodecanoyl)-L-homoserine lactone (3b), described in our manuscript (see Stacy, D. M.; Welsh, M. A.; Rather, P. N.; Blackwell, H. E. Attenuation of Quorum Sensing in the Pathogen Acinetobacter baumannii Using Non-native N-Acyl Homoserine Lactones. ACS Chem. Biol. 2012, 7, 1719-1728.). In our case, these AHLs were synthesized using CBS reduction methodology followed by HPLC purification to yield what we believed to be the pure diastereomers. During our biological characterization of these compounds, we found the AHLs to exhibit very similar autoinducer activities in Acinetobacter baumannii. In the report in ACS Chem. Biol., the authors prepared these same AHLs via Noyori reduction and in their hands, the 3b diastereomer exhibited a 40-fold decrease in autoinducer activity; thus, we sought to repeat our biological analyses to confirm our results. The diastereomeric AHLs prepared for our publication no longer existed in the laboratory and when we attempted to repeat the reported synthetic and purification methods, pure compounds could not be obtained, instead a mixture of 3a and 3b co-eluted as confirmed using chiral HPLC. Pure 3-hydroxy AHLs were obtained using the Noyori reduction conditions as reported by Blackwell and colleagues. To examine the effect of mixtures of 3a and 3b on autoinducer activity in Acinetobacter baumannii, we measured the EC50 values of pure AHLs prepared from the Noyori reduction methodology and varying mixtures of these diastereomers (1:1-1:9). Upon examination, little difference was found to exist between the EC50 values of pure 3a and a 1:1 mixture of 3a and 3b (0.173 mM and 0.498 mM, respectively; 2.9-fold difference). Moreover, our reported values of 0.67 mM and 0.82 mM for 3a and 3b, respectively, align with a ~1:1-1:2 mixture of the compounds. 

In the light of the results obtained, it is likely that the preparations employed for the work described in the PLOS ONE article included a mixture of the 3a and 3b diastereomers; the novel data do not support the conclusion that stereochemistry is not significant for the signaling activity of the two compounds.

The attached figure http://www.plosone.org/attachments/pone.0037102.comment1.pdf reports the results of our further analyses showing how the stereochemistry and the mixture of the two stereoisomers affect the biological activity. 

